# Phase IIb/III ATTENTION‐AD Study: Over Three Years of Continuous Treatment with Oral Blarcamesine Continues to Significantly Benefit Early Alzheimer’s Disease Patients

**DOI:** 10.1002/alz70859_101346

**Published:** 2025-12-25

**Authors:** Marwan N. Sabbagh, Stephen Macfarlane, Audrey Gabelle, Timo Grimmer, Kun Jin, William R. Chezem, Juan Carlos Lopez‐Talavera, Christopher U Missling

**Affiliations:** ^1^ Barrow Neurological Institute, Phoenix, AZ USA; ^2^ The Dementia Centre, HammondCare, St Leonards, NSW Australia; ^3^ Memory Resource and Research Center of Montpellier, CHU de Montpellier, Hôpital Gui de Chauliac, Montpellier France; ^4^ Technical University of Munich, School of Medicine and Health, TUM University Hospital, Center for Cognitive Disorders, Munich, Bavaria Germany; ^5^ Anavex Life Sciences Corp., New York, NY USA; ^6^ Anavex Life Sciences Corp, New York, NY USA; ^7^ Anavex, New York, NY USA

## Abstract

**Background:**

There are no approved oral disease‐modifying small molecule therapies for treatment of early Alzheimer’s disease (AD). Blarcamesine is an orally bioavailable small molecule that enhances autophagy through SIGMAR1 activation and restoration of cellular homeostasis in early AD.

**Method:**

The ATTENTION‐AD study was an up to 144‐week open‐label extension study subsequent the 48‐Week Phase IIb/III double‐blind placebo‐controlled ANAVEX^®^2‐73‐AD‐004 study, to evaluate the safety and efficacy of oral once daily blarcamesine in 508 participants with early AD. Delayed‐start analysis was performed to assess the effect of early treatment initiation up to 192 Weeks.

**Result:**

The ATTENTION‐AD trial confirmed the good efficacy and safety profile of once daily oral blarcamesine and also demonstrated the manageable nature of the most frequent treatment emergent adverse event (TEAE) (dizziness) observed in the preceding ANAVEX^®^2‐73‐AD‐004 trial, which was generally transient in duration (approx. 7‐11 days) and mild or moderate in severity (Grade 1 or 2). The titration schedule was adjusted to a slightly longer titration period in the ATTENTION‐AD trial, from previous 2‐3 weeks to 10 weeks. A markedly lower frequency of the TEAE of dizziness in the respective maintenance phase was observed: from previously 25.2% in the ANAVEX^®^2‐73‐AD‐004 trial to 9.6% in the ATTENTION‐AD trial, demonstrating the manageable nature of this TEAE. No severe or life‐threatening adverse events were attributed to blarcamesine. No Amyloid Related Imaging Abnormalities (ARIA) adverse events were identified. There were no deaths related to blarcamesine.

Efficacy of the cognitive and functional endpoints in the delayed‐start analysis of treatment resulted in significant outcomes, ADAS‐Cog13 (LS mean difference ‐3.83, P = 0.0165) and ADCS‐ADL (LS mean difference +4.30, P = 0.0206) at Week 192, reflecting importance of early treatment initiation with oral blarcamesine.

**Conclusion:**

Blarcamesine significantly reduced clinical decline showing meaningful benefit for early Alzheimer’s disease patients. Blarcamesine exhibited a favorable safety profile with no treatment‐related deaths and demonstrated no associated neuroimaging adverse events with continued treatment over 4 years. Altogether, these results indicate that blarcamesine may be an effective, safe, and novel scalable oral treatment for early AD.

